# Assessing the Diversity and Specificity of Two Freshwater Viral Communities through Metagenomics

**DOI:** 10.1371/journal.pone.0033641

**Published:** 2012-03-14

**Authors:** Simon Roux, Francois Enault, Agnès Robin, Viviane Ravet, Sébastien Personnic, Sébastien Theil, Jonathan Colombet, Télesphore Sime-Ngando, Didier Debroas

**Affiliations:** 1 Laboratoire “Microorganismes: Génome et Environnement”, Clermont Université, Université Blaise Pascal, Clermont-Ferrand, France; 2 CNRS, UMR 6023, LMGE, Aubière, France; Institute of Infectious Disease and Molecular Medicine, South Africa

## Abstract

Transitions between saline and fresh waters have been shown to be infrequent for microorganisms. Based on host-specific interactions, the presence of specific clades among hosts suggests the existence of freshwater-specific viral clades. Yet, little is known about the composition and diversity of the temperate freshwater viral communities, and even if freshwater lakes and marine waters harbor distinct clades for particular viral sub-families, this distinction remains to be demonstrated on a community scale.

To help identify the characteristics and potential specificities of freshwater viral communities, such communities from two lakes differing by their ecological parameters were studied through metagenomics. Both the cluster richness and the species richness of the Lake Bourget virome were significantly higher that those of the Lake Pavin, highlighting a trend similar to the one observed for microorganisms (i.e. the specie richness observed in mesotrophic lakes is greater than the one observed in oligotrophic lakes). Using 29 previously published viromes, the cluster richness was shown to vary between different environment types and appeared significantly higher in marine ecosystems than in other biomes. Furthermore, significant genetic similarity between viral communities of related environments was highlighted as freshwater, marine and hypersaline environments were separated from each other despite the vast geographical distances between sample locations within each of these biomes. An automated phylogeny procedure was then applied to marker genes of the major families of single-stranded (*Microviridae*, *Circoviridae*, *Nanoviridae*) and double-stranded (*Caudovirales*) DNA viruses. These phylogenetic analyses all spotlighted a very broad diversity and previously unknown clades undetectable by PCR analysis, clades that gathered sequences from the two lakes. Thus, the two freshwater viromes appear closely related, despite the significant ecological differences between the two lakes. Furthermore, freshwater viral communities appear genetically distinct from other aquatic ecosystems, demonstrating the specificity of freshwater viruses at a community scale for the first time.

## Introduction

Despite the large population sizes of microbes, their high reproductive rates and the potential for long-distance passive dispersal, an increasing amount of studies are showing that the transitions between marine and fresh waters are infrequent [1]. Indeed, marine and freshwater microbes are usually not closely related, often grouping into distinct marine and freshwater clades among bacteria [2] or eukaryotes [3]. Based on host-specific interactions, the presence of specific clades among hosts suggests the existence of freshwater-specific viral clades. Despite the paucity of molecular data from freshwater viruses, recent studies comparing freshwater and marine viruses have concluded on the existence of distinct clades [4], [5]. Nevertheless, these PCR-mediated analyses are restricted to chosen viral groups as no gene is universally conserved among viruses. In addition, part of the existing diversity of the viral groups studied is missed as PCR primers are based on previously known sequences described in public databases. Thus, both the diversity of freshwater viral communities and its distinction with marine viruses still need to be demonstrated on a community scale by studying not one but all the major viral families.

Viral metagenomics is a methodology capable of providing an exhaustive view of viral diversity [6], and it has so far revealed an important unknown diversity and an unexpected richness of viral communities [7]. Virome studies on freshwater environments were conducted on aquaculture facilities [8], and a polar lake in Antarctica [9], but never on temperate freshwater lakes. The viral diversity retrieved in these analyses was contrasted : aquaculture facilities were mainly composed of bacteriophages (*Myoviridae* and *Podoviridae*), whereas eukaryotic viruses, including phycodnaviruses and single-stranded DNA viruses accounted for a large proportion of the Antarctic viral communities. Thus, a fine-grained and exhaustive description of viral diversity in temperate freshwater lakes is needed to improve our knowledge about these communities and to offer the opportunity of identifying potential viral populations specific of those environments.

To assist in these goals, we performed a characterization of freshwater viromes from two french lakes: the lake Pavin and the lake Bourget which exhibit different trophic status, morphological and hydrological features (Table S1). Because species compositions and abundances of potential hosts have been shown to vary with lake trophic status, depth, watershed or size [3], [10], suggesting possible similar variations for viral communities, these two lakes are expected to be complementary systems for studies on freshwater viromes.

The two viromes were analyzed according to the following procedure : (i) the characteristics and richness indices of the two viral communities were determined, (ii) freshwater viromes were cross-compared to a set of previously published viromes in terms of sequence similarity and richness, (iii) the composition of the two communities were determined and phylogenetic analyses of the major families were computed in order to accurately describe the genetic diversity in these families.

## Results

### Overview of the two freshwater viromes

**Figure 1:**
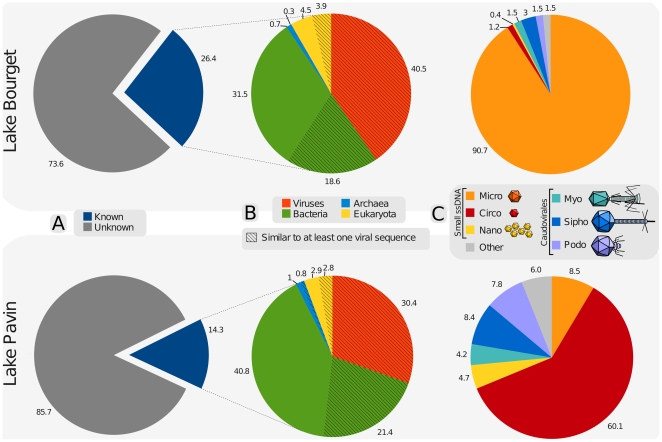
Composition and taxonomic affiliations of Lake Bourget and Lake Pavin virome reads as determined by similarity to known sequences. (A) The percent of “known” virome sequences when compared to the NR protein database. A read was considered “known” if it had a significant similarity in NR (BLASTx using thresholds of 10^−3^ on e-value and 50 on bit score). (B) Breakdown of the “known” sequences into Viruses, *Bacteria*, *Archaea*, or *Eukarya* using similarity results against NR. Hatched parts were reads having a best BLAST hit against a non-viral sequence, but still presenting significant similarity against a complete virus genome sequence of the RefseqVirus database (tBLASTx using thresholds of 10^−3^ on e-value and 50 on bit score) and thus designated as reads “similar to at least one viral sequence”. (C) Taxonomic composition at the viral family level of these reads “similar to at least one viral sequence” computed using the GAAS pipeline. The “Other” category pools families which represented less than 1% of the full virome sequences. The number of sequences represented in each chart are as follow : 593,084 ; 156 772 ; 95,905 for charts A, B and C of Lake Bourget virome, and 649,290 ; 92,834 ; 47,345 for the Lake Pavin virome.

After 454 pyrosequencing and data filtering, viromes of 593,084 and 649,290 reads with an average length of 420 bp were available for Lake Bourget and Lake Pavin, respectively. The proportion of reads similar to protein sequences of the non-redundant NCBI database (NR) were 26.4% and 14.3% for Lakes Bourget and Pavin, respectively (Figure 1A). These proportions of “known” reads (reads with a BLAST hit against NR) are among the highest compared to published viromes (range 1%–28% with an average of 6,3% for aquatic environments [9], [11]). Yet, as read length influences these proportions of “known” reads [12] and as our reads are 400 bp versus 100 to 250 bp in previous studies, a direct comparison of BLAST hit ratios is questionable, so the “known” fractions were also determined using reads randomly reduced to 100 bp. Using shorter reads, the “known” fractions dropped to 2.2% in Lake Bourget and to 0.7% in Lake Pavin, the one of the Lake Pavin being the lowest among aquatic viromes (Table S2).

Among these “known” fractions, a majority of reads (69.6% for Lake Bourget and 59.5% for Lake Pavin) was most similar to non-viral sequences (Figure 1B), whereas our viromes were not contaminated by bacteria : the absence of 16S rRNA in both samples was checked by PCR amplification and a BLAST search for 16S rRNA sequences, and only a paucity of viromes reads were found to be partly similar to ribosomal proteins (16 reads in Lake Bourget virome, 6 reads in Lake Pavin virome). This result is consistent with previous studies [7] and is thought to be an indication of both the lack of viral gene annotation and the horizontal gene transfers between viral and host genomes.

The taxonomic composition deduced from the bacterial best BLAST hits was not consistent with the previously published data on bacterial communities from Lakes Bourget and Pavin ([10], [13] ; Table S3). In the two lakes, members of phylum *Actinobacteria* are considered as dominant in the bacterial fraction, but scarcely retrieved in the viromes. Conversely, a large proportion of virome reads are associated to *Firmicutes* and *Gammaproteobacteria*, two groups considered as rare or even absent in the lakes.

Finally, the gene functions retrieved in the two viromes were unambiguously related to the viral life cycle, with a large proportion of genes related to DNA metabolism, DNA replication, and capsid assembly (Table S4). On the 30 most retrieved PFAM domain, only one (PF05792, Candida agglutinin-like protein) was never found in a viral genome. However, considering that this protein is involved in cell-cell interaction, its presence in viral genomes is rather logical.

### Cluster and species richness in the two freshwater viromes

The virome from the Lake Bourget has a higher number of different clusters, as 590,000 randomly chosen reads from each virome were grouped in 272,948 and 113,454 different clusters for Bourget and Pavin, respectively. This higher cluster richness observed in viruses from the Lake Bourget is also revealed by rarefaction curves, as the viral community from Lake Pavin was almost entirely contained in the metagenome, whereas that from Lake Bourget is far from being covered by the virome (Figure S1). The species richness, assessed using PHACCS [14], was estimated to 43,236 and 29,936 different virotypes in Lake Bourget and Lake Pavin, respectively.

#### Comparison of the richness of 31 viromes

**Figure 2:**
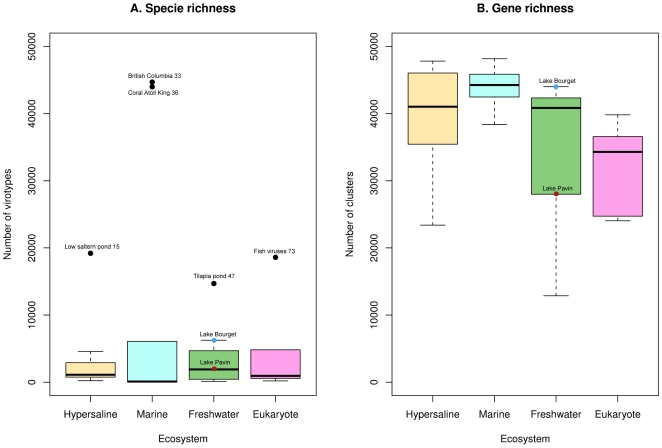
Boxplots of the estimation of species and cluster richness for viromes from different origins. Species richness (A) was estimated with PHACCS for virome subsamples (50,000 sequences, 100 bp). Cluster richness (B) was deduced from the number of different clusters formed from 50,000 input sequences of 100 bp. Viromes associated with extreme points are indicated on the plot, as well as the viromes from Lakes Bourget and Pavin.

Species richness (number of different virotypes) and cluster richness estimated from the two viromes were compared to the ones of 29 previously published viromes [9], [11] from seawater, freshwater, hypersaline and from viral communities associated with different eukaryotes (fish, coral and mosquito) (Table S2). Because numbers of reads of these 31 viromes were different, subsamples of 50,000 randomly-chosen 100-bp reads were generated in order to work with cross-comparable results. Number of virotypes was estimated using PHACCS [14], and plotted for each type of environment (Figure 2A). No significant link could be found between sample origin and virome species richness (one-way ANOVA: p-value = 0.542). The cluster richness of each virome, deduced from the read clustering (Figure 2B), was significantly different between the different environments (one-way ANOVA: p-value = 0.035). Genetic diversity of aquatic viromes (whether marine, freshwater, or hypersaline) was higher than that of viromes associated with eukaryotes, the highest diversity being observed in marine environment.

#### Similarity-based comparison between viromes

**Figure 3:**
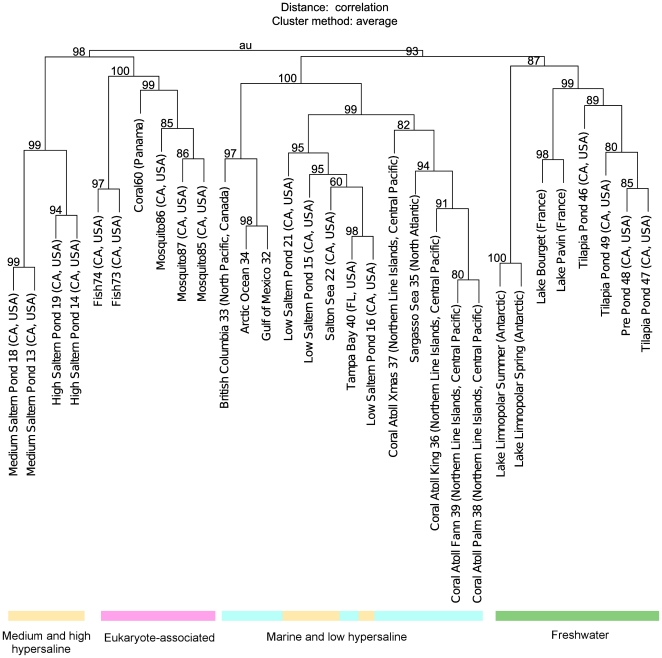
Virome hierarchical clustering tree based on sequence similarity. This tree was computed from tBLASTx comparisons of virome subsamples. Eukaryote-associated viromes were taken from fish, mosquito or coral samples. Hypersaline viromes are split into three categories based on salinity, as indicated in the original study of these viromes (http://www.theseed.org/DinsdaleSupplementalMaterial/).

Classically, bacterial metagenomes are compared using the taxonomic composition of their “known” fraction. Such a comparison would be misleading for viromes as their “known” fraction, generally lower than 10% of the reads, is not representative enough. Thus, the 31 viromes were not compared to a reference database but to each other in order to detect potential genetic links between the different viral communities. The hierarchical clustering tree, computed from tBLASTx comparisons, highlighted a separation of the viral communities according to four environment types: eukaryote-associated, high-salinity water (high and medium hypersaline), low-salinity water (seawater and low-hypersaline), and freshwater (Figure 3). Among freshwater viral communities, viromes from Lake Limnopolar were aggregated and distant from the other viromes, highlighting the specificity of these viral communities. Temperate freshwater viromes were also clustered, and split into two sub-groups: a group composed of the viromes from Lake Bourget and Lake Pavin and a group of viromes from aquaculture facilities [8].

### Composition of viral communities and subsequent phylogenies on the main identified viral families

#### General taxonomic composition

To characterize the composition of viral communities, reads similar to a known viral proteins were affiliated using best BLASTx hits against RefSeqVirus. These identified viral fractions represented 15% and 10 of the reads in Lake Bourget and Lake Pavin viromes, respectively (Figure 1C). The same viral families (Microviridae, Circoviridae, Nanoviridae, Myoviridae, Siphoviridae and Podoviridae) were retrieved in both lakes but in different relative proportions (Figure 1C). As previously reported in other ecosystems [9], [15], a high proportion of single-stranded DNA (ssDNA) viruses (Micro-, Circo- and Nanoviridae) virus was recorded.

#### Phylogenetic analysis of the main viral families

Using the procedure described by Roux et al. [16], phylogenetic analyses were performed on the major viral families of the two viromes using different marker genes. Virome reads homologous to each marker were assembled into contigs long enough to build informative phylogenetic trees for the targeted viral groups.

#### Phylogenetic analysis of small ssDNA viruses : Microviridae family

**Figure 4:**
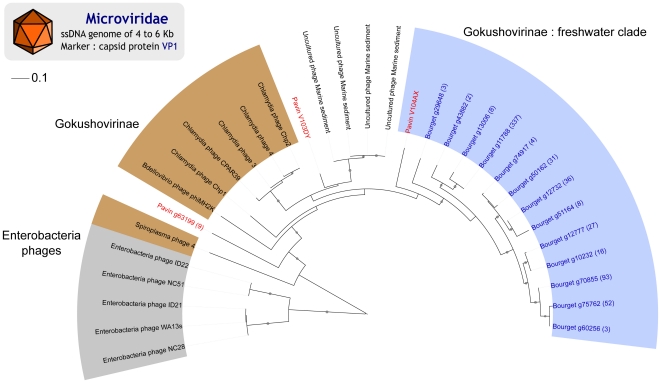
Maximum-likelihood tree for *Microviridae* (VP1). The main reference groups among *Microviridae* were retrieved and indicated on the tree (*Enterobacteria* phages in gray and *Gokushovirinae* in brown). The new group (*Gokushovirinae* : Freshwater clade) is colored in green. Leaf labels corresponding to virome sequences are colored (blue for Lake Bourget and red for Lake Pavin). The number of reads assembled is given in brackets for each contig. Nodes with at least 80% bootstrap support are flagged with black circles.

*Microviridae* form a group of ssDNA bacteriophages with a small capsid (30 nm), split into two sub-families: *Enterobacteria* phages, and *Gokushovirinae* [17], containing phages of *Chlamydiae*, *Bdellovibrio* and *Spiroplasma*. A set of 892 reads from Pavin and Bourget viromes, similar to the capsid protein VP1, was used to perform phylogenetic analyses. Metagenomic sequences were mainly affiliated to *Gokushovirinae* (78%) forming a clade related to *Chlamydiae* phages but distinct from references and with a high internal diversity, that we named “*Gokushovirinae* : Freshwater clade” (Figure 4). The other metagenomic sequences are distinct from both the *Enterobacteria* phages and the *Gokushovirinae* sub-families (22% of the reads).

#### Phylogenetic analysis of small ssDNA viruses: Circo- and Nanoviridae families

**Figure 5:**
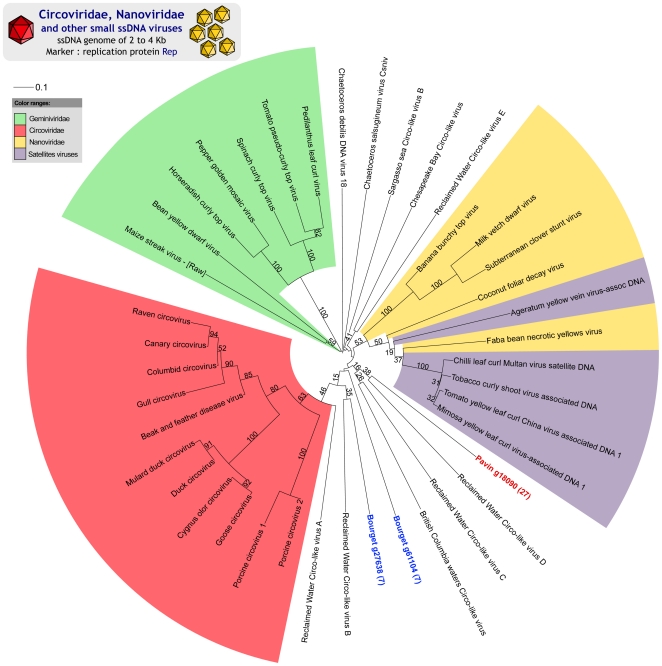
Maximum-likelihood tree for Circo-like viruses (Rep). This tree gathers sequences from four different viral families, i.e. *Circoviridae* in red, *Nanoviridae* in yellow, *Satellites viruses* in blue and *Geminiviridae* in green, sequences from two diatoms viruses (*Chaetoceros* viruses), sequences taken from marine and reclaimed water samples, and sequences from both the lakes studied here. Leaf labels corresponding to virome sequences are colored (blue for Lake Bourget and red for Lake Pavin). Bootstrap support is indicated for each node.

The replication protein Rep is conserved in different families of small ssDNA eukaryotic viruses (*Circoviridae*, *Nanoviridae*, Satellites viruses, *Chaetoceros* viruses and *Geminiviridae*) and in several recently-sequenced aquatic viruses described as “Circo-like” [18]. 223 assembled virome sequences were included in different part of the multiple alignment of the reference sequences of the Rep protein. Even if no close relationship was evident, sequences from Lake Bourget and Lake Pavin exhibited more similarities with Circo-like sequences from aquatic environments than with other ssDNA viruses. The length of branches in the resulting trees indicates high genetic distances between the different environmental sequences (Figure 5). These results shed light on a hitherto undescribed diversity for this viral family.

#### Phylogenetic analyses of dsDNA viruses: the Caudovirales

**Figure 6:**
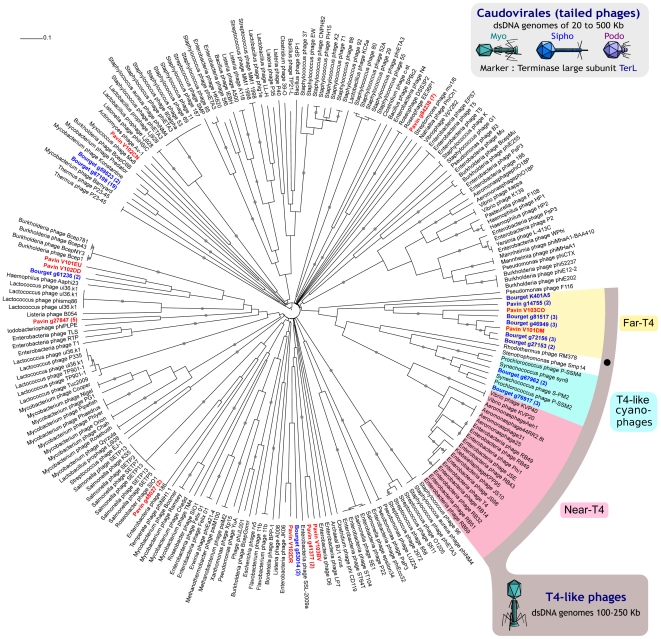
Maximum-likelihood tree for Caudovirales (TerL) : general view (A) and detailed view of the T4-like group (B). The main reference groups of T4-like phages are indicated (near-T4 in red, T4-like cyanophages in blue), and leaf labels corresponding to virome sequences are colored (blue for Lake Bourget and red for Lake Pavin). The Far-T4 group is highlighted in yellow. The number of reads assembled is given in brackets for each contig. Nodes with at least 80% bootstrap support are flagged with black circles. Rhodothermus RM378, the only cultured representative within the Far-T4 clade, is marked with a black dot.

Caudovirales form an order of dsDNA viruses better known as “tailed bacteriophages” whose diversity can be assessed with a gene coding for the large subunit of the terminase (TerL). The 185 virome sequences used in phylogenetic analyses were widely distributed among the *Myo-*, *Sipho-* and *Podoviridae* and were phylogenetically distant to known reference sequences, hightlighting an important uncharacterized diversity for Caudoviruses in freshwater environments (Figure 6).

**Figure 7:**
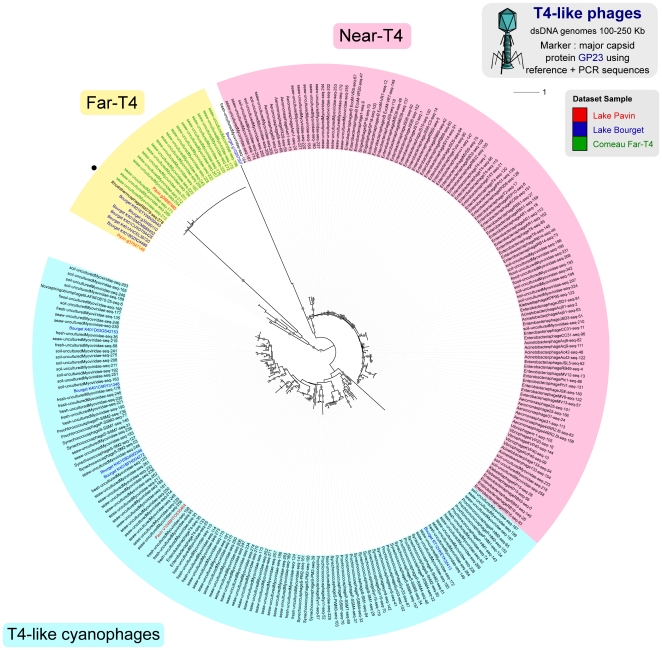
Maximum-likelihood tree for T4-like phage (GP23). The two main reference groups are indicated (near-T4 in red and T4-like cyanophages in blue). The Far-T4 group is highlighted in yellow. Leaves corresponding to virome sequences are colored (red for Lake Pavin and blue for Lake Bourget). Far-T4 sequences described by Comeau *et al.* are highlighted in green. Nodes with at least 80% bootstrap support are flagged with black circles. The sample origin of PCR-obtained sequences is designated on the leaf label (seaw stands for seawater, flood for floodwater, and fresh for freshwater). *Rhodothermus* RM378, the only cultured representative within the Far-T4 clade, is marked with a black dot.

A significant number of these sequences (20%) were related to T4-like phages (Figure 6) within the *Myoviridae* family. The diversity of this group has been previously explored using GP23 and G20 markers [19], [20] leading to the identification of different sub-groups: “Near-T4”, “T4-like cyanophages” and “Far-T4”, a group composed of only one sequenced genome (*Rhodothermus* phage RM378) and identified in marine waters by PCR approach on the GP23 gene [20]. According to G20-based phylogenetic analyses (Figure S2 and Figure S3), 11% of the 190 virome reads from Bourget and Pavin were affiliated to “T4-Like cyanophages” and 89% of these reads formed a new group including *Rhodothermus* phage RM378. Similar proportions were obtained with the GP23 marker, with 16% of the 251 reads affiliated to “T4-Like Cyanophage” and 84% forming a group containing the “Far-T4” group identified by Comeau et al. [20] (Figure S4 and Figure 7). Thus, freshwater virome sequences greatly expand the diversity of the previously identified Far-T4 group.

## Discussion

Viruses are the most abundant biological entities in fresh and marine waters, exceeding prokaryotic abundance 10-fold on average. They are important factors for the regulation of microbial community composition [21]–[23] and affect the cycling of carbon and nutrients [24], [25]. Yet, little is known about the composition and diversity of the temperate freshwater viral communities. This study examined such communities from two temperate freshwate lakes differing by their ecological parameters.

### Comparison of the two lacustrine communities

The fraction of known reads was higher in the Lake Bourget virome than in the Lake Pavin virome. When reduced to 100-bp reads, the Lake Pavin dataset was the virome with the lowest “known” fraction. These results highlight the lack of knowledge and reference sequences for viruses of these environments, especially for low trophic status waters. Genetic diversity appeared to be high in the Lake Bourget virome, as more than 600,000 reads were still not enough to cover the entire viral community gene pool for this sample. Even if this cluster richness is also high in the Lake Pavin virome, it appeared to be more than twofold lower than the cluster richness of the Bourget. The number of different virotypes (species richness) was also 44% higher in the virome of the Lake Bourget. Species compositions, diversities and abundances of potential hosts have been shown to vary with lake trophic status, depth, watershed or size [3], [10]. Moreover, the richness of bacterial and eukaryotic communities determined in different freshwater ecosystems were lower for low trophic status ecosystems such as Lake Pavin [3]. The analysis of the two viromes, highlighting a similar trend for two freshwater viral communities, corroborates the idea that viral diversity is correlate to the diversity of the organisms seen as potential hosts.

### Comparison of aquatic communities

The two freshwater viromes and 29 published viromes were compared through their gene and species richness and through their pairwise sequence similarities. These three analyses are particularly well adapted to viromes as they take into account not only the “known” reads but also the “unknown” reads, that make up the major share of the viral metagenomes in the present state of sequence databases. Species richness highlights the very broad genotypic diversity of viral communities, this richness remaining identical through the different ecosystems studied. Conversely, cluster richness was shown to vary between different environment types and appeared significantly higher in marine ecosystems. These differences could be linked to a difference in genome size (i.e. the same number of genomes would be retrieved in the different ecosystems, but their difference in sizes would lead to a difference in the number of clusters formed), or to a difference in gene and genome richness (in which case the difference in cluster numbers would be linked to a difference in the number of different viral genomes between the ecosystems). Furthermore, viral communities appear to hierarchically cluster according to salinity levels. Specifically, viromes from freshwater environments are clustered, reflecting significant genetic similarity between these viromes, despite the vast geographical distances between sample locations (Antarctica, North America and Europe). Marine environments, as well as hypersaline environments, were also gathered, revealing that viruses are notably distinct in the different aquatic environments. The differences between freshwater and saline aquatic viral communities presented here are consistent with specific studies led on single marker genes [1], [5] indicating that freshwater viral communities are specific and distinct from other aquatic viral communities. Moreover, considering that the viromes were not prepared in the same way (e.g. the use of CsCl gradient in some cases, PEG precipitation in others), the result that viromes cluster by salinity level is definitively reflecting a strong pattern of differentiation between aquatic viral communities.

### Small ssDNA viruses: an under-estimated group

A strong presence of ssDNA viruses (mostly *Microviridae* and *Circoviridae*) was found in viromes of Lake Bourget and Lake Pavin, both quantitatively and in terms of diversity. These viral groups have been previously identified in different aquatic ecosystems: an analysis of a Sargasso Sea virome described the presence of *Microviridae* in marine environments [26], and *Circoviridae* appeared to dominate Lake Limnopolar viral community [9]. The strong presence of small circular ssDNA viruses is generally thought to be a bias of the genomic amplification [27]. Indeed, small circular DNA fragments, such as genomes of *Circoviridae* and *Microviridae*, were shown to be preferentially amplified by the phi29 DNA amplification process needed to provide enough genetic material for pyrosequencing and used in all the viromes sequenced with NGS [9], [28]. However, even allowing for potential quantitative bias, *Microviridae* and *Circoviridae* are undoubtedly present in both Lake Bourget and Lake Pavin.

### Identification of a great diversity and of previously unknown clades

Our deep sequencing effort associated with a large read size made it possible to create direct phylogenetic analyses on marker genes from a virome. The main PCR pitfalls, namely the overriding need for a close known sequence in order to design PCR primers, could thus be circumvented. Analyses of the major viral groups found in the two communities all spotlight a very broad diversity and previously unknown virotypes. Indeed, new viral groups were highlighted for each studied family.

Using phylogenetic trees based on the replication protein, two studies recently reported new kinds of *Circoviruses* in aquatic environments that appear to be intermediate between the *Circoviridae*, *Geminiviridae* and *Nanoviridae*, with a very low similarity rate between these new sequences and the references [9], [18]. The same pattern emerges from the analysis of the Rep sequences retrieved in Lake Bourget and Lake Pavin. Since hosts of known *Circoviridae* are animals while those of known *Nanoviridae* and *Geminiviridae* are plants, potential hosts for the new viruses are difficult to assess.

A broad unknown diversity has also been found for *Microviridae* and *Caudovirales*, and putative freshwater clades could be described for these two families. According to our hypothesis, we expected to find freshwater-specific viral clades, due to the presence of typical freshwater clades for microorganism communities [2]. For *Microviridae*, a new group related to the *Chlamydiae* phage and including sequences from Lake Bourget and Lake Pavin is notably distinct from a group of uncultured *Microviridae* sampled from aquatic sedimentary structure [29]. Microphages communities from Lake Bourget and Lake Pavin thus appear to be closely related. These new viruses are unlikely *Chlamydiae* phages, since *Chlamydiae* are rarely detected in lakes and have never been retrieved in previous studies of Lake Bourget [13], [30], in the virome of which *Microviridae* were most abundant. Thus, it is likely that these new microphages infect another type of bacteria, or that the *Chlamydiae* phages host range is broader than previously thought. Otherwise, they could actually infect *Chlamydiae*, keeping their numbers below the detection threshold of classic diversity analysis protocols.

Phylogenetic trees drawn from TerL shed light on a high diversity among *Caudovirales*. The major share of the virome sequences is distributed far from references and far from each other, highlighting both the richness of Caudovirales freshwater communities and the absence of closely-related reference sequences.

In addition, some virome sequences appear to form a new clade related to the T4-like viruses, one of the most thoroughly described *Caudovirales* family. The use of degenerate primer sets for GP23 by Comeau *et al.* [20] recently highlighted a group of T4-like phages far from all references, designate as Far-T4 group. In our metagenomic data, phylogenetic analyses on marker genes all indicated the existence of a Far-T4 group, more than 80% of the T4-like phage sequences being affiliated to this group. A comparison of this new viral clade G20 sequences with known PCR primers revealed that these Far-T4 sequences would not be amplified by the primers commonly used to assess the diversity and distribution of T4-like phages in marine environments [31]. Furthermore, the analysis on GP23 shows that only a part of the diversity of the Far-T4 group is captured by the only primers amplifying DNA sequences from this group [20]. Moreover, this study proves that this Far-T4 group is not specific to marine ecosystems, despite the absence of amplification observed by Comeau *et al.* [20] on different freshwater samples. Thus, this study confirms undoubtedly the existence of a Far-T4 group as such viruses are retrieved using all three markers and indicates that this group is both greatly diversified and quantitatively important in freshwater ecosystems.

Finally, all the observed viral groups contained sequences from both lakes studied here, and phylogenetic trees revealed putative freshwater clades for *Microviridae* and *Caudovirales*, as was expected based on the specificity of microbial clades in lakes. Thus, phylogenetic analysis of major viral groups showed that the two freshwater communities are closely related, despite the significant ecological differences between the two lakes. These two viral communities are thus probably composed of evolutionarily close virotypes, and differ mainly in terms of the relative abundance of the viral species. The specificity of freshwater viruses, allready known for specific virotypes, is here demonstrated at a community scale and these results call for further studies of this kind on viral communities from a broad spectrum of environments.

## Materials and Methods

### Sample preparation

Samples were collected from Lake Bourget (45°43′47″N, 5°52′10″E) and Lake Pavin (45°29′41″N, 2°53′13″E) in July and June 2008. Lake Bourget and Lake Pavin are both freshwater lakes but they present significant differences in terms of biophysical parameters : Lake Bourget is ten times bigger than Lake Pavin, is deeper (145 m for Lake Bourget, 92 m for Lake Pavin), and has a greater drainage basin (56,000 ha vs 50 ha). Furthermore, Lake Bourget is a mesotrophic lake, exposed to human activity whereas the oligomesotrophic Lake Pavin is more isolated and located in a former caldera.

Both lakes were sampled by collecting 20 liters of water at a 5 m depth and running serial filtrations (25 µm, 1.2 µm, 0.2 µm). Virus-like particles (VLPs) were concentrated by tangential ultra-filtration (Amicon pump) followed by PEG precipitation [32]. Viral concentrates were then re-filtrated on a 0.2 µm screen, to remove any remaining cellular micro-organisms, then quantified by flow cytometry [33], [34]. Final concentration of viral particles was approximately 1.6*10^10^ VLPs/ml for both samples, which represented a concentration factor of 1000 from the initial concentration (about 10^7^ VLPs/ml for both samples). Viral concentrates were treated with DNAseI (Invitrogen) to remove external DNA fragments.

Encapsidated DNA was freed via thermal shock then purified using a QuiAmp DNA mini kit (Quiagen). To obtain sufficient genomic material for pyrosequencing, DNA amplification was run with a GenomiPhi Kit (GE Healthcare) which produced non-specific amplification through polymerase phi29 (as in [9], [28]). Absence of bacterial contamination was checked by flow cytometry [33] before DNA extraction. Potential bacterial contamination was also checked by amplifying the gene coding for 16S rRNA at each purification step (primer set 27f-1492r; [10]). No amplification was found for any of the samples. The two DNA preparations were subjected to a single pyrosequencing run by GATC Biotech (Germany) using a 454 Life Sciences GS-FLX Genome Sequencer.

The datasets generated for the Lake Bourget and the Lake Pavin were composed of 597,675 and 684,224 DNA sequences (i.e. reads) with a mean size of 433 bp and 412 bp, respectively. Replicate software [35] was used to remove exact duplicate reads, which accounted for 6% of the Lake Pavin virome and 1% of the Lake Bourget virome. Both viromes are available through the Short Read Archive under accession number ERP000339, and on the Metavir web-server ([16], http://metavir-meb.univ-bpclermont.fr; project “French lakes”).

### Public virome dataset

29 viromes available in public databases and composed of more than 50,000 sequences were downloaded for comparison with the viromes of Lakes Bourget and Pavin. 23 originated from aquatic environments and 6 were sampled from eukaryotes (Table S2). All viromes were screened for duplicate reads using Replicate [35]. In order to normalize the sequence size from these different datasets, we sampled 50,000 sequences of 100 bp for each.

### Viral communities cluster richness and rarefaction curves

The cluster richness of each virome subsample was assessed by clustering reads. Uclust [36] was used to cluster reads of each virome subsample at 75% identity. This clustering threshold was chosen based on the high divergence observed between viral genes, but similar results were obtained with thresholds of 90% and 98%. For each virome, a sub-sample was iteratively increased by 1.000 randomly selected sequences (without replacement) and clustered at each step using Uclust [36]. The number of clusters formed was plotted as a function of the number of input sequences. The viral cluster richness of the different types of environment were compared by a one-way ANOVA conducted using the R statistical software. For Lake Pavin and Bourget, these clusterings were also computed on whole viromes in order to draw rarefaction curves.

### Viral communities species richness

The species richness of each of virome subsamples was computed using the PHACCS tool ([14], http://biome.sdsu.edu/phaccs/), based on the contig spectrum obtained with a sequence assembly at 98% similarity on at least 35 bp, computation of all rank-abundance distribution laws and default parameters. The average genome size was determined using GAAS as in other studies [37]. The viral species richness of the different types of biomes were compared by a one-way ANOVA conducted using the R statistical software.

### Similarity-based comparison between viromes

Finally, we ran an *in silico* qualitative comparison between the different viromes based on sequence similarity (tBLASTx comparison) as described in [38] and [16]. Briefly, virome samples (50,000 sequences for each virome) are cross-compared to every other using tBLASTx. A similarity score is deduced, and used to hierarchically cluster viromes using the pvclust package of R software with default parameters [39].

### Taxonomic composition of the viromes

After removal of duplicate reads, virome sequences were analyzed without assembly and compared with BLASTx tool [40] against NR, NCBI's non-redundant amino acid sequence database. The best similarity for each virome read was parsed and assigned as “known” if there was a significant similarity to a protein from the NR database (thresholds of 10^−3^ on e-value and 50 on bit score) and else “unknown” (Figure 1A). In a second step, the reads from the “known” group were classified as viral, bacterial, archaeal, or eukaryotic based on their highest similarity (Figure 1B). Reads similar to provirus sequences are often similar to cellular organism sequences. To identify these transferred viral sequences, tBLASTx was used to compare the virome reads against the complete virus genome sequences of the RefseqVirus database. Any read with a significant similarity (thresholds of 10^−3^ on e-value and 50 on bit score) to one of the previous four taxonomic groups of NR that was also similar to a viral sequence of RefSeqVirus was counted as “similar to at least one viral sequence” (Figure 1B). These reads “similar to at least one viral sequence” were affiliated to viral families and the taxonomic composition of each virome was computed using the GAAS pipeline [26], with thresholds of 50% on similarity percent, 20% on query sequence length, and keeping only the top hit for each query (Figure 1C). 16S rDNA absence was checked via a BLASTn of the viromes reads against RDP [41], a 16S ribosomal DNA sequence database. The absence of bacterial contamination was confirmed by the very low number of reads presenting a best BLAST hit against ribosomal proteins (16 for Lake Bourget virome, 6 for Lake Pavin). The bacterial taxonomic composition was based on the reads which best BLAST hit against the NR database was a bacterial sequence. Functional annotations were deduced from a rpsBLAST against the PFAM database.

### Phylogenetic analysis of main viral families

Randomly sequenced metagenomic reads of around 400 bp do not cover the entire marker genes considered (as the markers considered here were between 600 and 1500 bp long). To circumvent this limitation, a custom-designed procedure was developed to automatically generate phylogenetic trees including metagenomic sequences for a marker gene of interest (Figure S5 ; markers used : VP1 for *Microviridae*, Rep for *Circoviridae*, *Nanoviridae* and *Geminiviridae*, TerL, GP23 and G20 for *Caudovirales*).

One marker gene was selected for each ssDNA viral group. For the *Microviridae* family, we used a gene coding for the major viral coat protein VP1, previously used as phylogenetic marker for these viruses [29]. For several other families of small eukaryotic viruses, including *Circoviridae*, *Nanoviridae*, and *Geminiviridae*, we chose the gene coding for a replication protein Rep previously described as a good marker [9], [42]. For dsDNA viruses, two types of markers were used : the broad-spectrum marker TerL, previously used to assess diversity among *Caudovirales*, including *Myoviridae*, *Siphoviridae*, and *Podoviridae* [43], and two well-known phylogenetic markers for T4-type phages, the major capsid protein gene GP23 and the capsid assembly protein gene G20 [19], [31], [44]. The two markers G20 and GP23 were used either with entire gene sequences from completely sequenced phages (Figure S2, Figure S4) or with shorter but more numerous PCR sequences as reference (Figure 7, Figure S3).

Briefly, metagenomic sequences homologous to each marker were retrieved via BLASTx against NR, and assembled using Cap3 [45] (98% identity on 35 bp) in order to have longer sequences at our disposal. Using these stringent assembly parameters makes it possible to group only sequences from the same virotype [14]. These sequences were aligned against a reference alignment, and alignment bounds for each metagenomic sequence were collected and used to define sub-alignements containing several metagenomic sequences. This step is useful to reduce the number of trees to be calculated and makes it possible to generate trees containing several metagenomic sequences. Multiple alignments were automatically curated using Gblocks [46] and the ten longest alignments were selected for each marker. Phylogenetic trees were generated using PhyML [47], with 100 bootstraps replicates. The trees used in the figures were manually edited using iTOL [48].

All these analyses can be viewed on-line on the Metavir web-server ([16], http://metavir-meb.univ-bpclermont.fr). Furthermore, the different Perl scripts designed for these analysis (Virome tBLASTx comparison and automatic tree generation), as well as the multiple alignments and phylogenetic trees generated for this study are available on demand.

## Supporting Information

Figure S1 **Rarefaction curves based on whole viromes.** Each virome was clusterized at 75% identity, and the curve presents the number of different clusters as a function of the number of input sequences.
(PDF)

Figure S2 **Maximum-likelihood tree for T4-like phages (G20).** The main reference groups are indicated on the tree (near-T4 in red, T4-like cyanophages in blue), and the Far-T4 group is highlighted in yellow. Leaf labels corresponding to virome sequences are colored (red for Lake Pavin and blue for Lake Bourget). The number of reads assembled is given in brackets for each contig. Nodes with at least 80% bootstrap support are flagged with black circles.
(PDF)

Figure S3 **Maximum-likelihood tree for T4-like phage (G20).** A phylogenetic tree has been drawn for the T4-like phage group, and the two main reference groups are indicated (near-T4 in red and T4-like cyanophages in blue). The Far-T4 group is highlighted in yellow. Leaf labels are colored according to their sample (red for Lake Pavin and blue for Lake Bourget). Nodes with at least 80% bootstrap support are flagged with black circles. The sample origin of PCR-obtained sequences are designated on the leaf label (seaw stands for seawater, flood for floodwater, and fresh for freshwater). Rhodothermus RM378, the only cultured representative within the Far-T4 clade, is marked with a black dot.
(PDF)

Figure S4 **Maximum-likelihood tree for T4-like phages (GP23).** The main reference groups are indicated on the tree (near-T4 in red, T4-like cyanophages in blue), and leaf labels corresponding to virome sequences are colored (red for Lake Pavin and blue for Lake Bourget). The Far-T4 group is highlighted in yellow. The number of reads assembled is given in brackets for each contig. Nodes with at least 80% bootstrap support are flagged with black circles.
(PDF)

Figure S5 **Schematic representation of the phylogenetic tree creation pipeline.**
(PDF)

Table S1 **Characteristics of the two lakes studied.**
(DOC)

Table S2 **Main characteristics of viromes included in the different comparisons.** Extraction methodology is indicated where available. Peg = polyethylene glycol; CsCl = Cesium Chloride. The BLAST hit ratio is the percentage of reads significantly similar to a protein of the database the non-redundant database (threshold of 10^-3^ on e-value and 50 on scores). As sequence comparison results depend on the length of the sequences, reads were reduced to 100-bp long for all viromes.
(DOC)

Table S3 **Bacterial taxonomic composition as deduced from virome reads best BLAST hits, compared with previously published data.** These previous data are from a metagenome for Lake Bourget, and from 16SrRNA PCR amplification for Lake Pavin.
(DOC)

Table S4 **Main functions retrieved in the viromes.** In the first table, the 30 most retrieved PFAM domains in the viromes are listed, with the number of sequences for each virome alongside informations about their description in viral genomes, or the fact that most of the sequences from this domain are of viral origin (identified as «viral» domains). In the second table, the 30 most retrieved GO terms are listed, with the associated number of sequences for each virome.
(DOC)
