# Supplementary material for: Assessing the Diversity and Specificity of Two Freshwater Viral Communities through Metagenomics
Source: PLoS One. 2012 Mar 14;7(3):e33641. doi: 10.1371/journal.pone.0033641 (PMC3303852; doi:10.1371/journal.pone.0033641)
Supplement: Figure S1 — Rarefaction curves based on whole viromes. Each virome was clusterized at 75% identity, and the curve presents the number of different clusters as a function of the number of input sequences. (PDF) [file pone.0033641.s001.pdf]

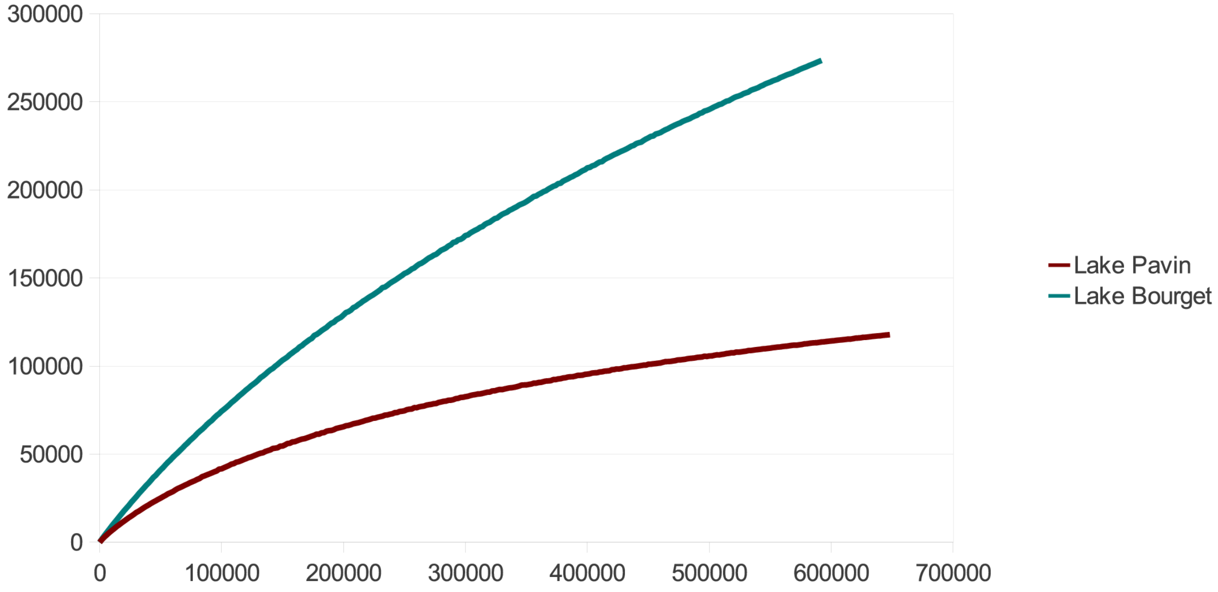

Figure S1. Rarefaction curves based on whole viromes. Each virome was clusterized at 75% identity, and the curve presents the number of different clusters as a function of the number of input sequences.
