# Supplementary material for: Assessing the Diversity and Specificity of Two Freshwater Viral Communities through Metagenomics
Source: PLoS One. 2012 Mar 14;7(3):e33641. doi: 10.1371/journal.pone.0033641 (PMC3303852; doi:10.1371/journal.pone.0033641)
Supplement: Figure S2 — Maximum-likelihood tree for T4-like phages (G20). The main reference groups are indicated on the tree (near-T4 in red, T4-like cyanophages in blue), and the Far-T4 group is highlighted in yellow. Leaf labels corresponding to virome sequences are colored (red for Lake Pavin and blue for Lake Bourget). The number of reads assembled is given in brackets for each contig. Nodes with at least 80% bootstrap support are flagged with black circles. (PDF) [file pone.0033641.s002.pdf]

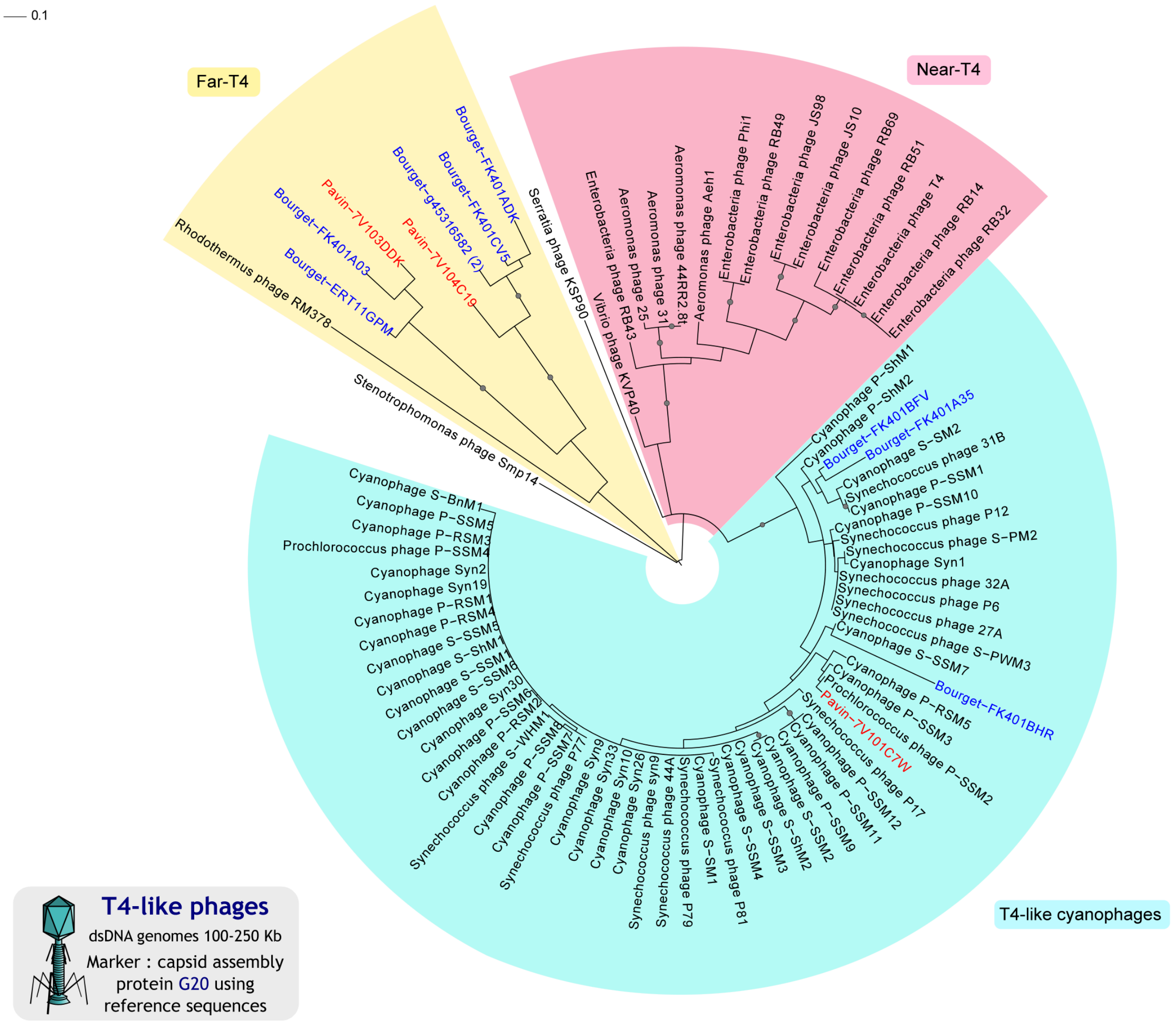

Figure S2. Maximum-likelihood tree for T4-like phages (G20). The main reference groups are indicated on the tree (near-T4 in red, T4-like cyanophages in blue), and the Far-T4 group is highlighted in yellow. Leaf labels corresponding to virome sequences are colored (red for Lake Pavin and blue for Lake Bourget). The number of reads assembled is given in brackets for each contig. Nodes with at least 80% bootstrap support are flagged with black circles.
