# Supplementary material for: Assessing the Diversity and Specificity of Two Freshwater Viral Communities through Metagenomics
Source: PLoS One. 2012 Mar 14;7(3):e33641. doi: 10.1371/journal.pone.0033641 (PMC3303852; doi:10.1371/journal.pone.0033641)
Supplement: Figure S3 — Maximum-likelihood tree for T4-like phage (G20). A phylogenetic tree has been drawn for the T4-like phage group, and the two main reference groups are indicated (near-T4 in red and T4-like cyanophages in blue). The Far-T4 group is highlighted in yellow. Leaf labels are colored according to their sample (red for Lake Pavin and blue for Lake Bourget). Nodes with at least 80% bootstrap support are flagged with black circles. The sample origin of PCR-obtained sequences are designated on the leaf label (seaw stands for seawater, flood for floodwater, and fresh for freshwater). Rhodothermus RM378, the only cultured representative within the Far-T4 clade, is marked with a black dot. (PDF) [file pone.0033641.s003.pdf]

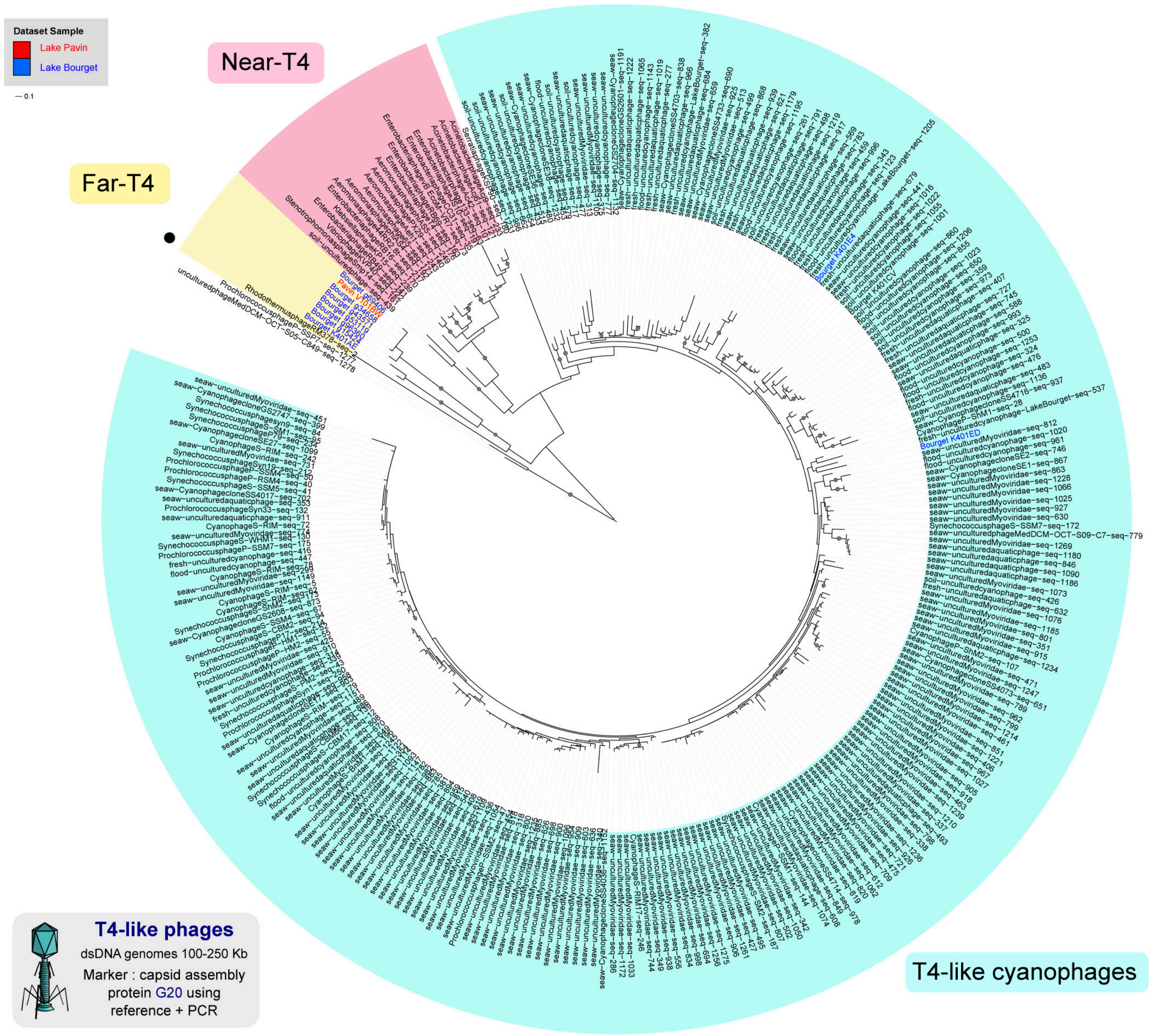

Figure S3 . Maximum-likelihood tree for T4-like phage (G20). A phylogenetic tree has been drawn for the T4-like phage group, and the two main reference groups are indicated (near-T4 in red and T4-like cyanophages in blue). The Far-T4 group is highlighted in yellow. Leaf labels are colored according to their sample (red for Lake Pavin and blue for Lake Bourget). Nodes with at least 80% bootstrap support are flagged with black circles. The sample origin of PCR-obtained sequences are designated on the leaf label (seaw stands for seawater, flood for floodwater, and fresh for freshwater). Rhodothermus RM378, the only cultured representative within the Far-T4 clade, is marked with a black dot.
