# Supplementary material for: Assessing the Diversity and Specificity of Two Freshwater Viral Communities through Metagenomics
Source: PLoS One. 2012 Mar 14;7(3):e33641. doi: 10.1371/journal.pone.0033641 (PMC3303852; doi:10.1371/journal.pone.0033641)
Supplement: Figure S4 — Maximum-likelihood tree for T4-like phages (GP23). The main reference groups are indicated on the tree (near-T4 in red, T4-like cyanophages in blue), and leaf labels corresponding to virome sequences are colored (red for Lake Pavin and blue for Lake Bourget). The Far-T4 group is highlighted in yellow. The number of reads assembled is given in brackets for each contig. Nodes with at least 80% bootstrap support are flagged with black circles. (PDF) [file pone.0033641.s004.pdf]

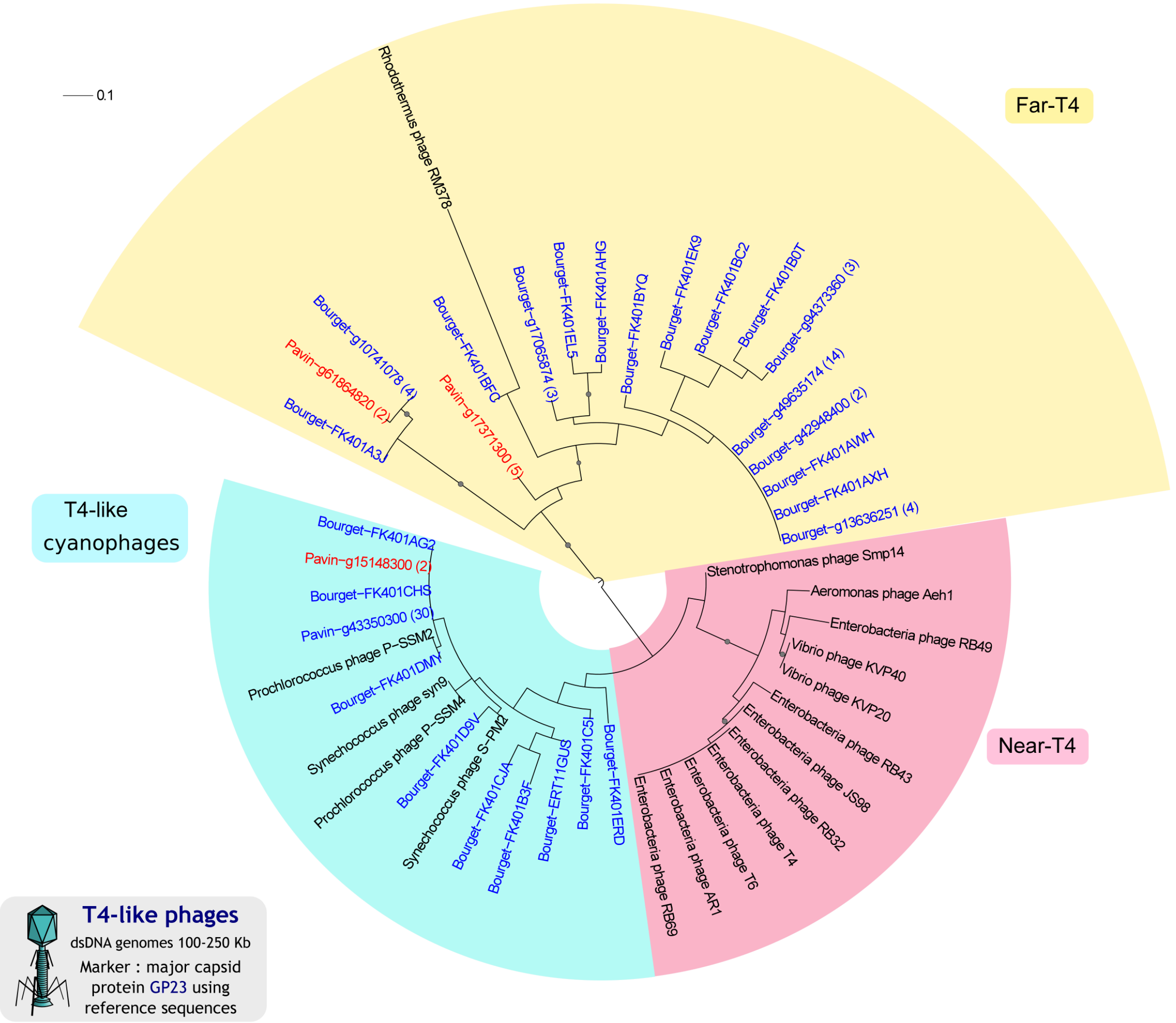

Figure S4. Maximum-likelihood tree for T4-like phages (GP23). The main reference groups are indicated on the tree (near-T4 in red, T4-like cyanophages in blue), and leaf labels corresponding to virome sequences are colored (red for Lake Pavin and blue for Lake Bourget). The Far-T4 group is highlighted in yellow. The number of reads assembled is given in brackets for each contig. Nodes with at least 80% bootstrap support are flagged with black circles.
