# Supplementary figures and images for: Assessing the Diversity and Specificity of Two Freshwater Viral Communities through Metagenomics
Source: PLoS One. 2012 Mar 14;7(3):e33641. doi: 10.1371/journal.pone.0033641 (PMC3303852; doi:10.1371/journal.pone.0033641)

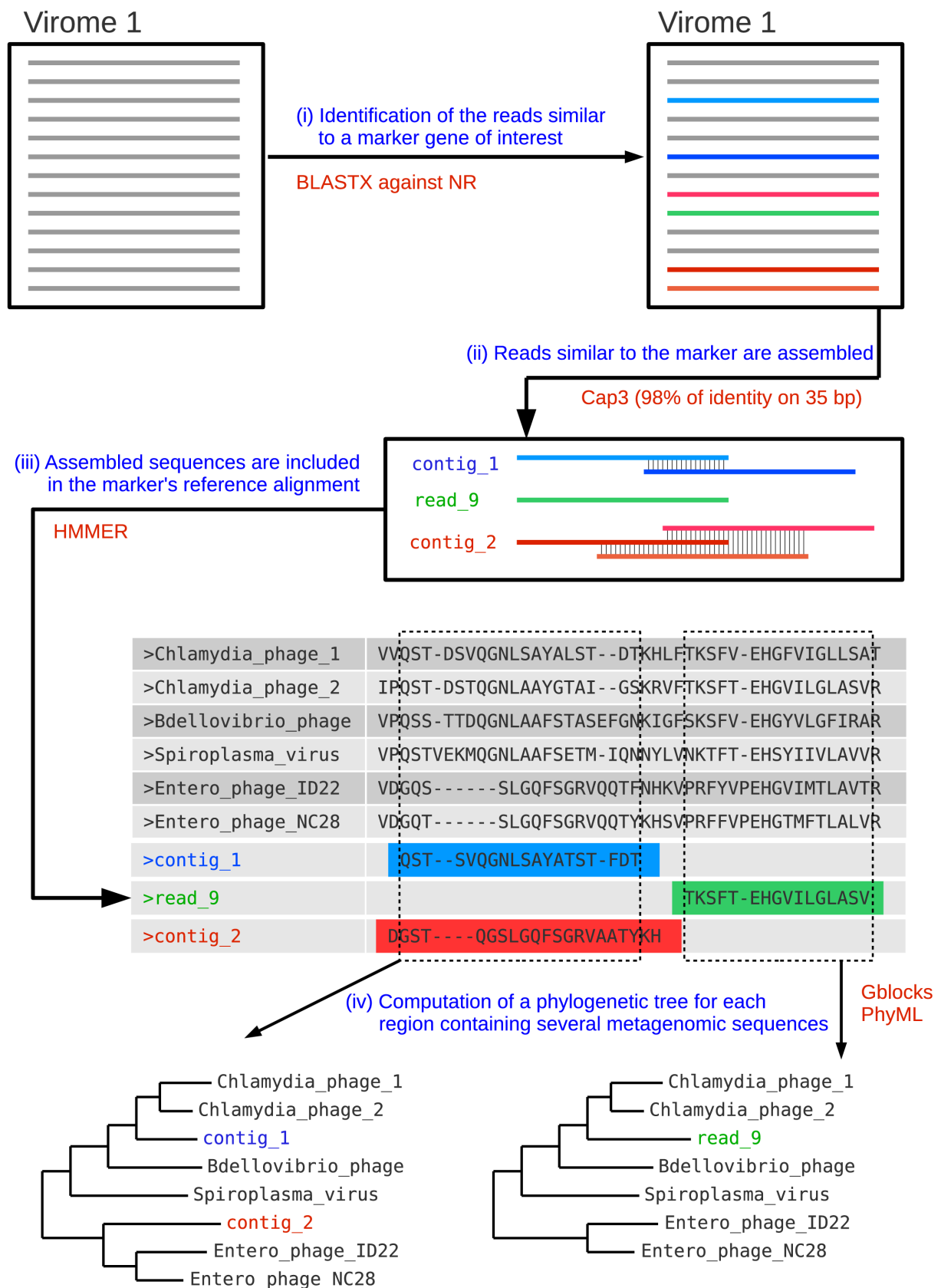

**Figure S5. Schematic representation of the phylogenetic tree creation pipeline.**

Supplement: Figure S5 — Schematic representation of the phylogenetic tree creation pipeline. (PDF) [file pone.0033641.s005.pdf]
