# Supplementary material for: Assessing the Diversity and Specificity of Two Freshwater Viral Communities through Metagenomics
Source: PLoS One. 2012 Mar 14;7(3):e33641. doi: 10.1371/journal.pone.0033641 (PMC3303852; doi:10.1371/journal.pone.0033641)
Supplement: Table S1 — Characteristics of the two lakes studied. (DOC) [file pone.0033641.s006.doc]

Table S1. Characteristics of the two lakes studied.
