# Supplementary material for: Assessing the Diversity and Specificity of Two Freshwater Viral Communities through Metagenomics
Source: PLoS One. 2012 Mar 14;7(3):e33641. doi: 10.1371/journal.pone.0033641 (PMC3303852; doi:10.1371/journal.pone.0033641)
Supplement: Table S2 — Main characteristics of viromes included in the different comparisons. Extraction methodology is indicated where available. Peg = polyethylene glycol; CsCl = Cesium Chloride. The BLAST hit ratio is the percentage of reads significantly similar to a protein of the database the non-redundant database (threshold of 10-3 on e-value and 50 on scores). As sequence comparison results depend on the length of the sequences, reads were reduced to 100-bp long for all viromes. (DOC) [file pone.0033641.s007.doc]

Table S2. Main characteristics of viromes included in the different comparisons.

Extraction methodology is indicated where available. Peg = polyethylene glycol; CsCl = Cesium Chloride. The BLAST hit ratio is the percentage of reads significantly similar to a protein of the database the non-redundant database (threshold of 10^-3^ on e-value and 50 on scores). As sequence comparison results depend on the length of the sequences, reads were reduced to 100-bp long for all viromes.
