# Supplementary material for: Assessing the Diversity and Specificity of Two Freshwater Viral Communities through Metagenomics
Source: PLoS One. 2012 Mar 14;7(3):e33641. doi: 10.1371/journal.pone.0033641 (PMC3303852; doi:10.1371/journal.pone.0033641)
Supplement: Table S3 — Bacterial taxonomic composition as deduced from virome reads best BLAST hits, compared with previously published data. These previous data are from a metagenome for Lake Bourget, and from 16SrRNA PCR amplification for Lake Pavin. (DOC) [file pone.0033641.s008.doc]

Table S3. Bacterial taxonomic composition as deduced from virome reads best BLAST hits, compared with previously published data.

These previous data are from a metagenome for Lake Bourget, and from 16SrRNA PCR amplification for Lake Pavin.
