# Supplementary material for: Assessing the Diversity and Specificity of Two Freshwater Viral Communities through Metagenomics
Source: PLoS One. 2012 Mar 14;7(3):e33641. doi: 10.1371/journal.pone.0033641 (PMC3303852; doi:10.1371/journal.pone.0033641)
Supplement: Table S4 — Main functions retrieved in the viromes. In the first table, the 30 most retrieved PFAM domains in the viromes are listed, with the number of sequences for each virome alongside informations about their description in viral genomes, or the fact that most of the sequences from this domain are of viral origin (identified as «viral» domains). In the second table, the 30 most retrieved GO terms are listed, with the associated number of sequences for each virome. (DOC) [file pone.0033641.s009.doc]

Table S4. Main functions retrieved in the viromes.

In the first table, the 30 most retrieved PFAM domains in the viromes are listed, with the number of sequences for each virome alongside informations about their description in viral genomes, or the fact that most of the sequences from this domain are of viral origin (identified as « viral » domains). In the second table, the 30 most retrieved GO terms are listed, with the associated number of sequences for each virome.
